# Toward Unbiased High-Quality Portraits through Latent-Space Evaluation

**DOI:** 10.3390/jimaging10070157

**Published:** 2024-06-28

**Authors:** Doaa Almhaithawi, Alessandro Bellini, Tania Cerquitelli

**Affiliations:** 1 Department of Control and Computer Engineering, Politecnico di Torino, 10129 Torino, Italy; tania.cerquitelli@polito.it; 2 Prime Lab, Mathema s.r.l., 50142 Florence, Italy; abel@mathema.com

**Keywords:** latent space density, industrial survey, Isometric Mapping (ISOMAP), StyleGAN2, model bias, qualitative and quantitative analysis, dimensionality reduction

## Abstract

Images, texts, voices, and signals can be synthesized by latent spaces in a multidimensional vector, which can be explored without the hurdles of noise or other interfering factors. In this paper, we present a practical use case that demonstrates the power of latent space in exploring complex realities such as image space. We focus on DaVinciFace, an AI-based system that explores the StyleGAN2 space to create a high-quality portrait for anyone in the style of the Renaissance genius Leonardo da Vinci. The user enters one of their portraits and receives the corresponding Da Vinci-style portrait as an output. Since most of Da Vinci’s artworks depict young and beautiful women (e.g., “La Belle Ferroniere”, “Beatrice de’ Benci”), we investigate the ability of DaVinciFace to account for other social categorizations, including gender, race, and age. The experimental results evaluate the effectiveness of our methodology on 1158 portraits acting on the vector representations of the latent space to produce high-quality portraits that retain the facial features of the subject’s social categories, and conclude that sparser vectors have a greater effect on these features. To objectively evaluate and quantify our results, we solicited human feedback via a crowd-sourcing campaign. Analysis of the human feedback showed a high tolerance for the loss of important identity features in the resulting portraits when the Da Vinci style is more pronounced, with some exceptions, including Africanized individuals.

## 1. Introduction

Machine Learning is mainly seen as a method of computing approximate functions that link data (input) with a label (output) to solve tasks that previously could not be solved by a human or a traditional algorithm. Deep learning has shifted the focus from approximate functions to latent spaces and how the approximate functions embed the data into these spaces through their parameters. Tuning the parameters of the approximate functions (e.g., approximating the model weights) is conducted by training the model, which is driven by the loss function of the task calculated from the labels. However, from an application perspective, the real goal is to create a latent space of data representation that provides a numerical/vectorial representation that effectively supports a particular machine learning task.

Although the meaning of *latent* is “existing but not yet manifest”, *latent space* is dependent on the context in which the term is used in different domains. Latent space generally refers to a space that is not tangible but is inferred or explored from the observed data. However, in machine learning (ML), *latent space* refers to a multidimensional numerical space (usually lower-dimensional than the original data space) that models the data under analysis.

More specifically, each layer within an ML model learns how to build its own latent space to represent or embed the data while retaining as much information about the data structure as possible. These spaces, once created, can later be used to explore the data properties and the underlying geometry of the manifold on which the data lives [1]. Much of the research has focused on investigating how exactly such a geometry represents the original data space (cf. [2,3,4,5]); others directly investigate the relationship between the latent data representation (embeddings) to obtain a better model of the data distribution (cf. [1,6,7]). However, both approaches have been used in various applications, e.g., anomaly detection (cf. [8]), data augmentation (cf. [9]) and data manipulation (cf. [10]), explainability (cf. [11]) as well as text and image generation (cf. [12,13]).

Recently, many large language models (LLMs) (e.g., generative pre-trained Transformers (GPTs) [14]) have used a latent space in which the relationships between words (tokens) are very precisely tuned to a variety of different contexts. Previously, image-based latent representations have been extensively studied. The leading examples are generative adversarial networks (GANs) ([15]) and variational autoencoders (VAEs) [16], where the former have demonstrated a high capability in generating and creating non-existent but realistic data, while the latter are still superior in terms of computational and time costs. In any case, the exploration and interpretation of such latent spaces are not trivial.

Although latent spaces embed real-world data in a low-dimensional space, this space is usually not low-dimensional enough to be easily analyzed (e.g., GPT has an embedding vector with 12,288 dimensions and the latent space of StyleGAN2 consists of 18×512); thus, *dimensionality reduction* techniques are required in such cases to better highlight the underlying data relationships.

In this work, we address a social-specific challenge in the DaVinciFace application (described in Section 3.1), namely to generate a Leonardo da Vinci-style portrait for each person. In our study, we analyze the ability of DaVinciFace to correctly perform portrait generation in terms of gender, age, and race. To this end, we thoroughly analyze the latent image space of StyleGAN2 on a dataset of 1158 human face images by first applying a dimensionality reduction technique (namely ISOMAP [17]) to visualize the dataset and investigate the density of latent vectors. Our study highlights the behavioral differences between sparse and dense representations of human features. We then present the detailed results of an industrial survey we sent to users of the application, which highlights potential biases related to gender and race.

The main contributions of this paper are the following:

1. The extension of DaVinciFace portraits to evaluate its capability to generate portraits for different social categories in the style of the Renaissance genius Leonardo da Vinci, and the demonstration of the effectiveness of latent space to support this goal.
2. A qualitative and quantitative analysis of a significant number of portraits to provide clear evidence of the effectiveness of our methodology in using DaVinciFace to create high-quality and realistic portraits in terms of diversity of facial features (e.g., beard, hair color, and skin tone).
3. Analyzing user feedback collected via a survey on the performance of DaVinciFace using a scale of identity vs style trade-off settings, including subjects with diversity regarding gender, race, and age. Where a high tolerance for the loss of identity features is observed in general to preserve more style features.

The remainder of this paper is organized as follows: The literature review is discussed in Section 2, the presentation of our methodology is discussed in Section 3, the obtained results are discussed in Section 4, and the discussion and conclusion are discussed in Section 5 and Section 6, respectively.

## 2. Related Work

Here, we discuss the current state of research on the use of artificial intelligence (AI) in art and its acceptance in the community of artists and non-artists. We then explain the latent spaces and dimensionality reduction techniques and possible forms of biases in AI models.

*Artificial intelligence for art: Visual AI applications for art generation and style transfer*. With the unprecedented success of AI-based solutions in almost all areas of life, art is no exception. AI is used not only for analysis, authorship identification, or forgery detection but also for creative generation and style transfer (cf. “Creating Art with AI” [18], “AI art in architecture” [19], and “Can Computers Be Creative” [20]). Recently, many AI-based art applications have emerged, but not all have been well received by critics or even non-experts; therefore, some critical studies and reviews have been developed (cf. [21,22,23,24]). The style transfer approach is an active field of research not only in art (cf. [25,26]), but also in text processing [27], medical cancer classification [28], and in videos [29]. In this paper, we investigate the main features of an existing application that creates Da Vinci-style portraits by conducting an online survey among the users of the application, distinguishing art-related backgrounds, gender, and age.

*Latent spaces and density.* A latent space is mainly concerned with how the model layers represent the data in it. Therefore, studying the characteristics of this space is crucial in most real-world applications. By learning such characteristics, tasks such as classification, prediction, or even generation become clearer and easier [1]. The most studied latent spaces are those generated by either generative adversarial networks (GANs) [15] or variational autoencoders (VAEs) [16]. However, GAN-based spaces are not only used intensively for the generation and manipulation in computer vision applications ([13,30]), but also for medical data, sensors, multi-modal data, and others (cf. medical image synthesis [10], brain imaging [31], collocating clothes [32], cross-modal image generation [33], and a built environment [34]). For example, the latent space StyleGAN2, first proposed in [35] and improved in [36], is one of the most widely used pre-trained models for generating realistic faces from noise, for which its ability to learn unsupervised high-level attribute separation (e.g., pose) [37] has been demonstrated. StyleGAN usually requires task-specific training for different tasks, but in terms of image manipulation and editing tasks, it produces high-quality and realistic generations, which has encouraged many researchers to propose tools to detect the generated fake photos to limit misuse and forgery (cf. [38,39]). In this work, we dive deep into the latent space of StyleGAN2 to visualize, analyze, and observe how the representations of the data in this space are either sparse or dense.

*Dimensionality reduction*. Although GAN-based frameworks reduce the dimensionality of high-dimensional input data to their latent spaces [12], the dimensions of these spaces are not low enough to be analyzed by humans (e.g., the latent space of StyleGAN2 is 18×512 [36]). Some research has proposed to apply clustering and data exploration techniques within the latent representation to better understand these spaces and disentangle the original data features (cf. embedding algorithm [40], attribute editing and disentanglement [41], clustering [6], interpretability and disentanglement [42], latent space organization [43], and disentanglement inference [44]); others use traditional non-linear *dimensionality reduction* (cf. [25,45]). Non-linear dimensionality reduction techniques are used for numerous purposes, e.g., for feature extraction [46], data visualization [47], pattern recognition [48] or even as a pre-processing step [49]. Isometric mapping (ISOMAP), discussed in [17], is one of the timeless algorithms of nonlinear projection-based algorithms that focus on global structure. More recent algorithms preserve more information in the reduced dimensions when the local geometry is close to Euclidean geometry, such as t-distributed stochastic neighbor embedding (t-SNE) [50] and uniform manifold approximation and projection (UMAP) (cf. [51,52]). In this work, we use ISOMAP to visualize the latent space of StyleGAN2 as it can understand the global structure of the data.

*Bias of AI models (in human images)*. Biases in AI [53] can generally be due to either a bias in the data or in the model processing. The latter is not easy to detect as the decision of the model is not readable by humans, making the detection and characterization of bias challenging. The bias in the dataset can be caused by the labeling of the data, which is a subjective task [54]. The bias of the model in photo-based systems and its mitigation are studied in depth in face recognition with an in-depth analysis of bias related to gender or race (cf. [53,54,55,56,57,58]). However, in style transfer applications (see [59,60,61]), a different type of bias occurs. This bias may be related to the photos of the reference style, and so far the style features cannot be completely separated from the subject presented in the reference to be transferred without compromising the identity of the new subject. In this paper, we investigate bias in the application of DaVinciFace focusing on gender and race aspects (see Section 4.3 and Section 4.4 for our analysis).

*Exploring latent space for artistic or human face applications*. In [62], the researchers implemented DeepIE (deep interactive evolutionary) with the style-based generator of a StyleGAN model to generate visual art from the fusion of two original works, and they collected subjective ratings through a questionnaire. However, they were concerned with visual art in general and did not focus on human portraits or the model’s bias toward social categories. On the other hand, the work of [63] develops a tunable algorithm to mitigate the hidden biases in the training data of human faces in a variational autoencoder-based model, while our analysis deals with GAN-based models for artistic applications. Ref. [64] investigates the entanglement problem using the InterFaceGAN framework on StyleGAN2 to improve the quality of the synthesized images, while our work focuses on the artistic application DaVinciFaceand the specific features concerning social categories. The idea of analyzing the effect of latent spatial representations in preserving specific features of human faces, especially the beard, for use in artistic portraits has been preliminary introduced before in [25]. The methodology outlined in this paper constitutes a substantial advancement from [25], introducing: (1) the setup process for configuring the application to create Da Vinci-style portraits, (2) the establishment of a pipeline for gathering human feedback alongside its corresponding subjective evaluation, (3) an extensive experimental assessment aimed at comprehending how latent representations encapsulate portrait features, with a nuanced focus on examining socially specific challenges concerning race, age, and gender, and (4) the execution of a crowd-sourcing survey to collect feedback regarding DaVinciFace’s capacity to generate high-quality portraits within an artistic framework.

## 3. Methodology

In this section, we present the materials and methods we used in our research. The methodological approach is characterized by: (1) First, we describe the main components of the application DaVinciFace (detailed in Section 3.1), an existing application used to generate a Da Vinci-style portrait. Then, (2) we describe how we used its encoder and decoder in exploring the StyleGAN2 latent space to find better configurations and settings for the mixture between the subject and the style photos (detailed in Section 3.2), resulting in a collection of different possible settings. Finally, (3) we evaluate these settings through a crowd-sourcing survey to collect human feedback. In doing so, we ask for feedback on the output generated by the application with different celebrities and test cases as subject inputs (detailed in Section 3.3).

### 3.1. DaVinciFace Application

**Figure 1:**
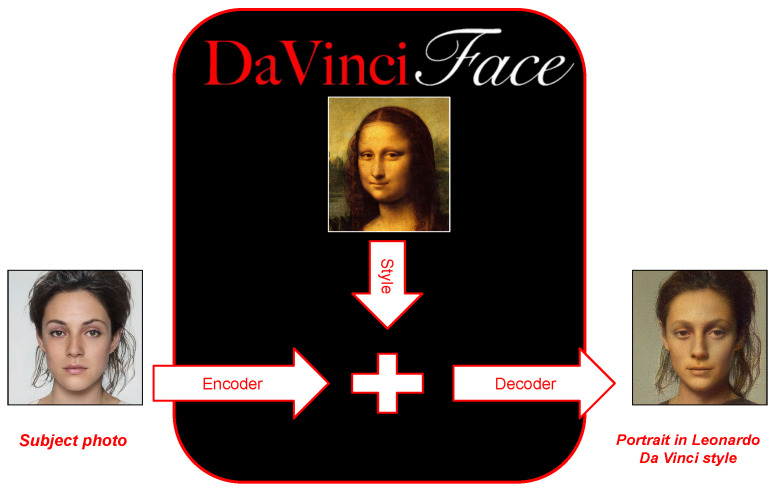
DaVinciFace. Main components—general view.

DaVinciFace (is a software registered with the SIAE (Italian Authors and Publishers Association)—www.davinciface.com) is a system developed by Mathema—an innovative SME (small- to medium-sized enterprise) based in Florence, Italy—DaVinciFace is a software registered with the Italian Authors and Publishers Association (SIAE) developed by Mathema s.r.l. (an innovative SME based in Florence, Italy) and available online: www.davinciface.com (accessed on 27 June 2024). DaVinciFace aims to create a portrait in the style of Leonardo da Vinci from a photograph of a human face. The main steps, shown in Figure 1, are as follows: (1) Projecting the photograph of the person’s face into the latent space using the latent space of StyleGAN2 [36]. (2) Blending with the latent vector of the style reference photo. (3) Generate a photo of the resulting vector showing a Da Vinci-style portrait of the person.

**Figure 2:**
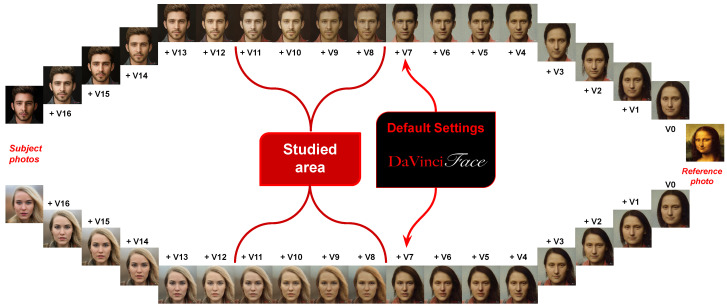
Two examples showing the possible settings when blending two image representations in the StyleGAN2 latent space (the subject and the reference image), highlighting the default settings of DaVinciFace and the area of the study.

*StyleGAN2 latent space*, proposed in [36], is an improved version of StyleGAN [35] that maps the input photo to a latent space *W* with 18 different vectors with 512 features each. As described in [35], the first four vectors correspond to higher-level aspects such as pose, general hairstyle, face shape, and glasses, while the colors of eyes and hair, lighting, or finer facial features are not modeled. The second four vectors model smaller facial features, hairstyle, and open/closed eyes, and finally the remaining vectors mainly provide color scheme and microstructure. Although StyleGAN’s latent space is considered disentangled and produces high-quality photos, this disentanglement is not yet complete in terms of the individual vectors. Figure 2 shows two subject examples with all possible combinations of blending their latent representations with the representation of the reference image generated by DaVinciFace.

*In* DaVinciFace *default settings*, led by [35], the first 8 vectors must necessarily come from the subject. The DaVinciFace application, therefore, takes the first eight vectors from the subject, while the remaining vectors come from the style photo (see the default settings in Figure 2). Table 1 shows six subjects with their portraits using these default settings. During the course of using the application, some users commented that the self-portraits they created did not look like their original photo and that they could not consider the portrait as their own, which drew attention to the trade-off between style and identity preservation in such an application. In this paper, we focus on the middle vectors from the 9th to the 12th vectors (from vector 8 to vector 11, starting from 0, see the examined area in Figure 2) to further investigate the entanglement of face- and identity-specific features. The methodology described in Section 3.2 makes it possible to identify the most effective configurations that can be set in DaVinciFace, while the survey (described in Section 3.3) made it possible to identify the best image among them according to the users.

### 3.2. Parameter Setting

**Figure 3:**
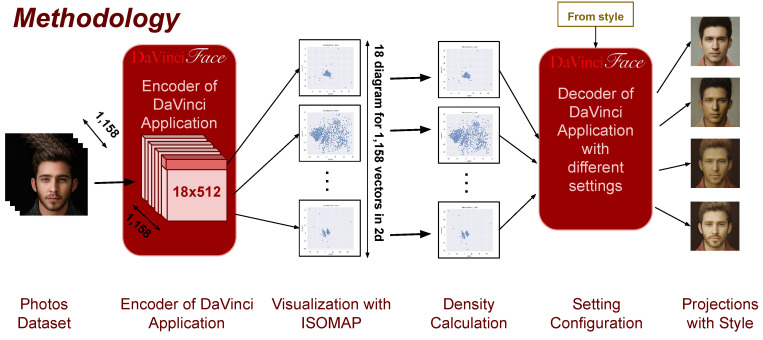
The methodology for selecting the most effective configurations to generate Da Vinci style portraits.

Figure 3 shows our steps for selecting the most effective configurations for creating Da Vinci-style portraits with respect to the different social categories of the subject. To investigate and explore the latent space of StyleGAN2, we collected and manually labeled a dataset of 1158 photos (detailed in Section 4.1), which includes 744 (64.25%) male and 414 (35.75%) female subjects. We then used the DaVinciFace encoder to project them into the latent space of StyleGAN2. We examined the latent representations (consisting of 18 vectors, each representing 512 features) of these photos by first visualizing each vector using a nonlinear projection technique (namely ISOMAP) and then computing the density of the resulting two-dimensional spaces. We found a large variation in the vector distributions, especially in the density, with some vectors being significantly sparser than others. Choosing different configurations to include either sparse or dense vectors, and projecting the photos of mixing led by these configurations using the DaVinciFace decoder, show the higher effect of sparse vectors on identity preservation.

### 3.3. Survey Design

**Figure 4:**
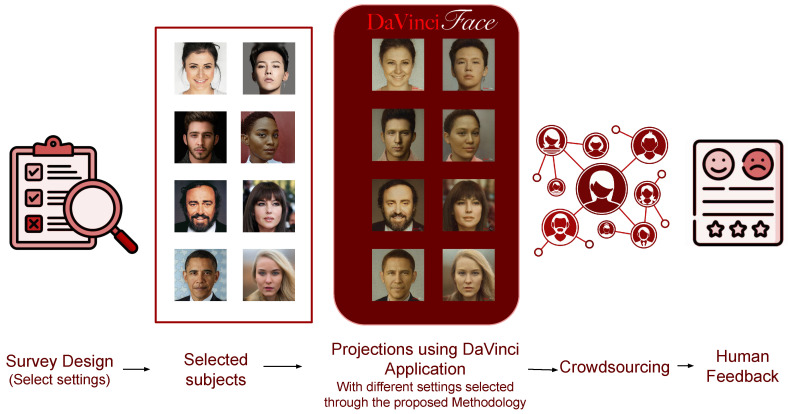
The pipeline for capturing human feedback and conducting a subjective evaluation.

Since the evaluation of the preservation of style and identity features is very subjective, it requires a subjective evaluation approach. To this end, we rely on a survey tool that allows us to collect human opinions and evaluate some specific aspects to quantify the key aspects of the data-driven applications. Collecting subjective opinions could lead to a more objective view following the method discussed in [22,24]. We conducted a crowd-sourcing evaluation (see Figure 4) by designing and launching a survey that was sent to DaVinciFace users to collect their subjective feedback. The aim of the survey was to collect both quantitative and qualitative data to answer the following questions:

**RQ1.**  *Is the current version of* DaVinciFace *able to sufficiently preserve the identity of the test subject?*

**RQ2.**  *Is there a better trade-off between style and identity preservation than the current version of* DaVinciFace *to provide a better portrait?*

Figure 4 shows different settings for mixing the subject and the style vectors in order to embed different ratios of identity and style features and we generated the corresponding Da Vinci-style portraits. We selected celebrities and non-celebrities with different characteristics in terms of gender, race, and age. And then, we promoted the survey and gathered feedback from 360 users.

**Figure 5:**
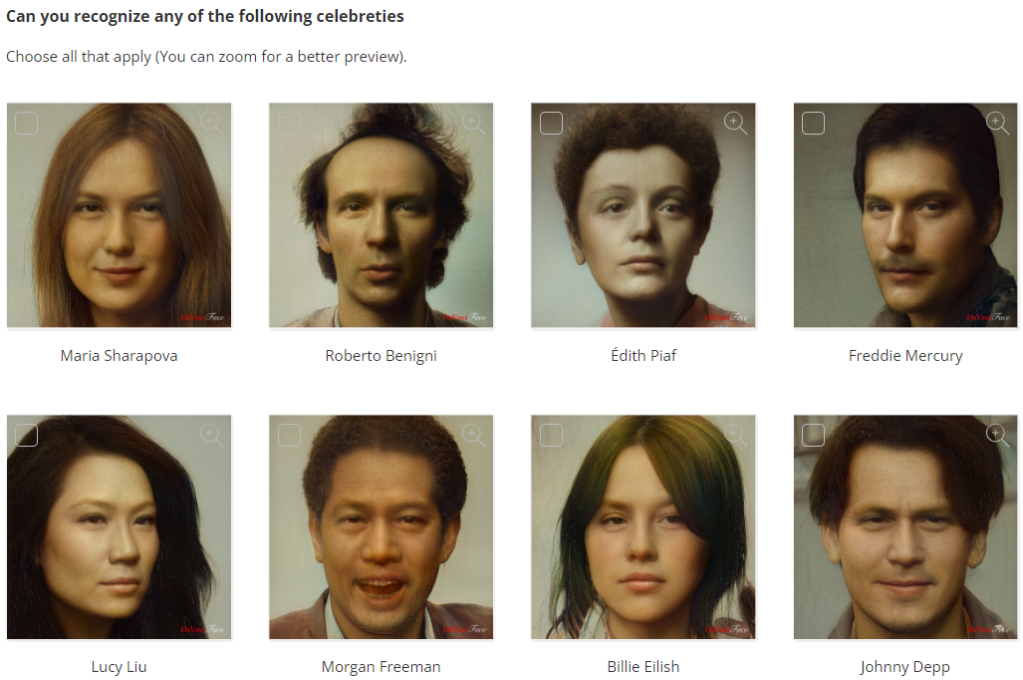
The survey question to address RQ1, where users were asked to select all recognized celebrities from eight Da Vinci portraits of (starting from top left) Maria Sharapova, Roberto Benigni, Edith Piaf, Freddie Mercury, Lucy Liu, Morgan Freeman, Billie Eilish, Johnny Depp. The celebrities were selected to cover different social categories, nationalities, and fields of activity.

**Figure 6:**
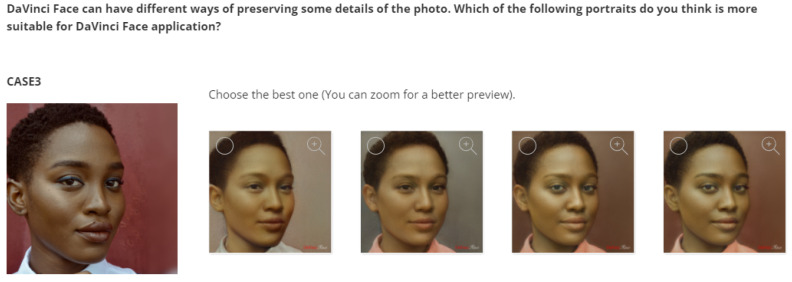
Examples of the 8 questions to address RQ2, which include test case 3, starting with the original photo, and then 4 different options with different settings that gradually increase the identity as the style preservation decreases.

*Survey Content*. To answer the above research questions, the survey included the following:

1. To address RQ1, the survey presents portraits of celebrities with the default settings and asks participants to select those they can recognize. By asking the user to select all recognized celebrity portraits, we can measure identity preservation in the default settings. However, the use of celebrities in a particular domain may influence the recognition of the celebrity itself. We selected eight celebrities from different fields and social characteristics, namely Maria Sharapova, Roberto Benigni, Edith Piaf, Freddie Mercury, Lucy Liu, Morgan Freeman, Billie Eilish, and Johnny Depp (see Figure 5).
2. To address RQ2, the survey presents different portraits created with different settings for the selected subjects, using the original image as a reference. By asking the user to select the best portraits, we can identify the best settings for the trade-off between style and identity preservation. We selected eight subjects (see Table 2), including four different celebrities with different fields and social characteristics (namely Monica Bellucci, Luciano Pavarotti, G-Dragon, and Barack Obama) and four other test cases (an example is shown in Figure 6).

*Survey Design Validation*. With the ultimate goal of designing and distributing an effective survey (similar to the authors in [65,66]), we evaluated the survey content with the support and feedback of a group of 19 domain experts. In detail:

- Instrumentation: In designing this survey, we conducted a pilot survey with 19 participants to help us determine the extent to which the questionnaire was understandable and complete. Participants had the opportunity to give their feedback on the questionnaire in terms of wording, clarity, and presentation.
- Selection of participants: Participants took part in the survey voluntarily.
- Maturation: Risks of fatigue or boredom were not considered as the average completion time was 3:44 min.
- The representativeness of the participant population was ensured by sending the survey to all users of the DaVinciFace.

## 4. Results

In this section, we discuss our experimental results to better understand how the latent representations embed the characteristics of the analyzed data, to address social-specific challenges related to racial, age, and gender diversity. To this end, we first describe the dataset and the corresponding latent representations in the Section 4.1 and Section 4.2. The discussion of the effects of some representations on the projected portraits (generated with the application DaVinciFace) in terms of gender and other characteristics is presented in Section 4.3, while the analysis of the crowd-sourcing survey to collect feedback in an artistic context is answered in Section 4.4.

### 4.1. Dataset and Latent Representations

We used a dataset of 1158 input images from the test environment of the application DaVinciFace with the corresponding latent vectors (18×512 each). The dataset is protected by copyright and, therefore, cannot be published. However, it consists of images of faces used in the first steps of creating and testing the application. The image is pre-processed with the two most important steps before projection:

- Human face detection: using a pre-trained model on the FFHQ dataset available on paperswithcode.com/dataset/ffhq (accessed on 14 May 2022) that extracts the most distinct face in the image;
- Centering and cropping the detected face in a square frame with the dimensions 1024×1024.

The projection into the latent space of the pre-trained model StyleGAN2 is performed in reverse order, starting with a random latent vector, generating the image, calculating the pixel-wise loss between this image and the original, and optimizing the latent vector, which is repeated for 1000 iterations. The output for each image consists of 18 vectors, each with a length of 512. For all results reported in this paper, we normalized the mixed vector in the latent space before creating the image, and we used a variance proportion of 0.4%.

### 4.2. Dimensionality Reduction and Density Calculation

We used ISOMAP to visualize each vector distribution in two-dimensional space to examine the effects of each vector—in terms of its density—on the output to detect any disentanglement between the images under study and their representation in latent space. Table 3 and Table 4 show the ISOMAP representations of the 18 vectors of the latent vectors of the points in the dataset using the scatter plot and the kernel density estimation plot, respectively (count starts at zero). The differences in the distributions can be clearly seen in Table 3. Some vectors are sparser than others (e.g., vectors 2 and 15), others are very dense around zero (e.g., vectors 5 and 6).

Furthermore, Table 4 shows that the density within the sparse vectors is not uniform. While vector 2 has approximately one central dense region, vector 15 has 3 regions. Within these vectors, we found that the coarse features (the first 5 vectors) and the fine features (the last six vectors) are sparser, while the middle vectors are relatively dense (except for vector 8), suggesting that sparse vectors may entangle more distinguishable features, while dense vectors embed the general human features. This motivates us to further investigate the effects of sparse and dense vectors on the creation of the resulting image. Since ISOMAP tends to learn the manifold of the original data and preserve the geometry [49], this gives us evidence that the features in the StyleGAN2 space not only have a local geometry but also a global geometry.

To obtain a quantitative measure of the density of the vectors, we calculate the average distance between the data points, as shown in Table 5. First, we reduce the dimensions of the individual vectors from 512 to two each using ISOMAP and then calculate the average Euclidean distance between the resulting two-dimensional representations. Table 5 underpins the previous discussion, so we can see the large difference in the average distance between the sparse and dense vectors (the minimum is vector 6 at 5.42 and the maximum is vector 3 at 49.73).

The full disentanglement of these vectors is still ongoing and the analysis is mainly based on the results discussed in [35], where the initial vectors are for pose and coarse features, gradually moving to fine features and style. However, gender, race, and age are distributed throughout the vectors as they are based on different facial and style features.

### 4.3. The Effect of Sparse Vectors

To better understand the effect of vectors on human facial features, we show the images resulting from blending the subject image with the style image using different settings and analyze the difference. As mentioned in Section 3.1, we focus on the middle vectors from vector 8 to vector 11 to investigate the social-specific feature entanglement.

Compared to the default settings of DaVinciFace which can be seen in Table 1, bearded or mustachioed males obtain less or almost no bearded/mustachioed portraits, and in general all examples lose important identity features such as eyebrow shape, cheekbones, and chin structure as well as lip and nose type. In particular, the square face shape, the very light eyebrows, the double lower eyelid, the Greek nose, and the corners of the mouth are strongly influenced by the reference photo of Da Vinci’s masterpiece, the famous Mona Lisa. This has also been commented on, in rare cases, by users of the application who noted the lack of self-representation in the resulting portraits, which can be self-referential and subjective. However, we reduce self-involvement by using the subjects (celebrities or not) to provide subjective feedback, but with some self-detachment in the judgment.

Table 6 compares six examples, starting with the subject image on the left, then the portrait with the default settings (vectors 0 to 7 from the subject image and the rest from the style image), and then continuing to the right, with each time adding another vector from the subject image instead of the style image (using the same subjects from Table 1). The effect of vector 8 is immediately apparent in the identity and gender features such as the beard and mustache in the male portraits and hair color and makeup in the female portraits.

However, adding more vectors (to the right) has a slight effect on increasing the identity features, and the style is gradually lost as the colors are lightened and changed. In addition, not only are the identity features clearer in the male portraits, but the individuals also tend to become younger toward the right. In the female examples, on the other hand, the light eye and hair color in the second example are more clearly visible, as is the light skin color in the sixth example and the dark skin of the fourth example. Our original aim is to enhance the identity while retaining the Da Vinci style as much as possible.

If we focus more on the effect of vector 8, it is expected to embed more distinctive features due to its low density compared to the other vectors (as explained in Section 4.2). Table 7 shows the two test cases (cases 1 and 2) and another five examples of bearded and blond subjects. In the first row, the first 12 vectors of the subject, including vector 8, are compared (with the same settings as in the last column Table 6), and in the second row, the same settings are kept, but vector 8 from the style image is used instead. The absence of this vector has a significant effect on the hair color and beard, but also on the chin, face shape, and eye type. However, other features such as eyebrow shape, nose type, and lips are not as strongly affected. We conclude that this sparse vector has a greater influence on identity and gender features than the other dense vectors. However, it is not the only one.

### 4.4. Survey Results

**Figure 7:**
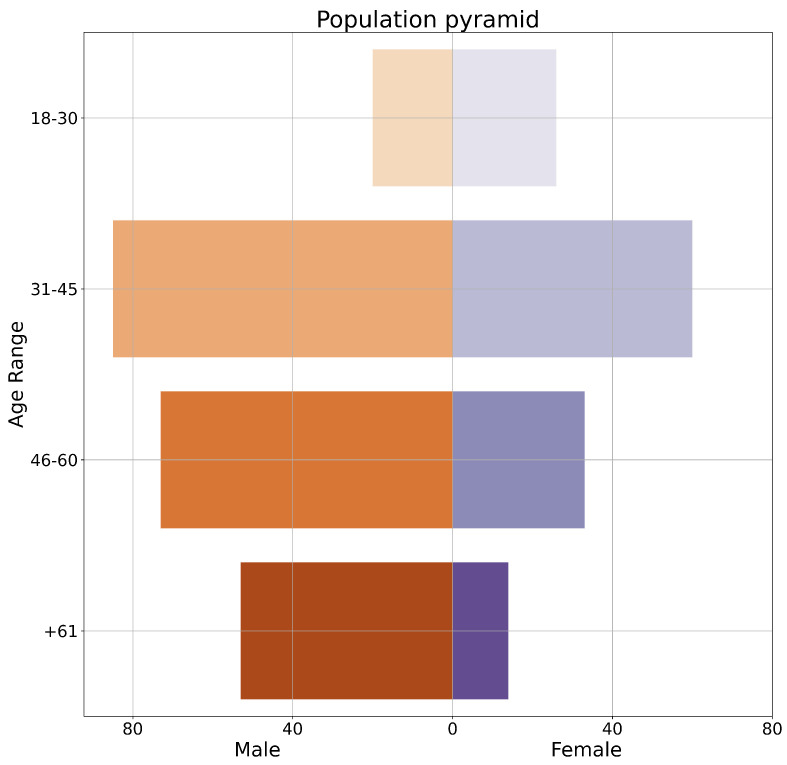
Population pyramid of participants shows the number of male (in orange) and female (in purple) participants for each age group.

We evaluate our results using a crowd-sourcing survey. The survey was advertised for one month, conducted online, and sent to all users of the application. Out of 525 total views, 370 completed the survey and 9 started it without completing it. This results in a participation rate of 72.2% and a completion rate of 97.6%, with an average completion time of 3:44 min. We used SurveyHero (www.surveyhero.com (accessed on 27 June 2024) is a software for designing, collecting, and analyzing survey responses) to design the survey and collect the responses. Figure 7 shows the population pyramid of participants in terms of age range and gender, male in orange and female in purple.

The demographic and background information on the participants is as follows:

- Gender perspective, out of 360 responses: 224 (62.22%) are men, 129 (35.83%) are women and 7 (1.94%) preferred not to answer.
- Age perspective: Out of 360 responses, 39.44% of participants belong to the age group (31–45), followed by 28.89% in the age group (46–60), 18.33% are older than 61 and 13.33% are younger than 30.
- Art-related background: Most users are interested in art (56.82%), followed by people not related or are not interested in art (21.17%), while professional artists and art students represent 16.71% and 5.29% respectively.

**Figure 8:**
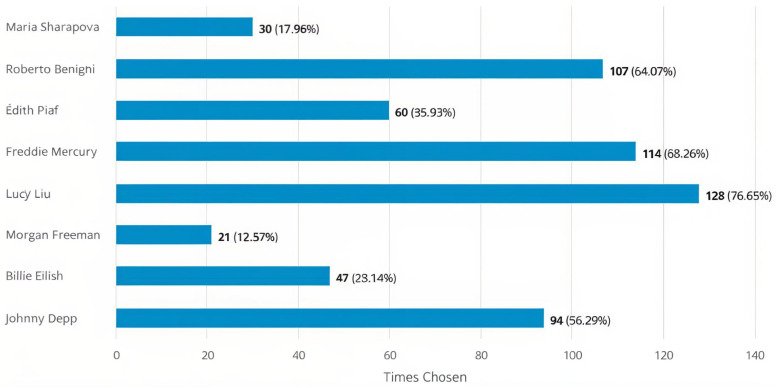
RQ 1: Number of positive responses (preserving the identity of the test subjects) for each selected test subject. The percentage indicates the number of users who recognized the celebrity divided by the total number of responses to this question (the total number of responses was 167).

The results of the survey on research question 1 (RQ1) (whether the default setting sufficiently preserves the subject’s identity) are shown in Figure 8, which shows that the highest recognition was given to Lucy Liu, followed by Freddie Mercury and then Roberto Benigni.

In order to address research question 2 (RQ2) mentioned in Section 3.3 (i.e., finding a better trade-off between style and identity preservation than the default settings), the survey contained 8 questions presenting four different celebrities (i.e., Monica Bellucci, Luciano Pavarotti, G-Dragon, and Barack Obama) and four additional test cases (see Table 2). Each question contains the photo of the corresponding subject with the following four settings: (a) default settings; (b) add vector 8 from the original image; (c) add up to vector 10 from the original image; (d) add up to vector 11 from the original image; (see Figure 6 as an example). The user can select the most favorable alternative from the various alternatives.

Table 8 shows the statistics of the selected options for the 8 subjects. According to the responses, the default version (which has the most style) is chosen most often, although identity preservation is the least favorable. More specifically, the percentage of default settings increases when the subject is a brunette female (Monica Bellucci with 67.93% and test case 4 with 57.49%), while it decreases for bearded males (test case 1 with 31.21% and Luciano Pavarotti with 43.84%). However, the default settings are no longer the preferred option if the test subject is not Caucasian (G-Dragon with a majority of 38.15% for option (b), Barack Obama with a majority of 28.86% for option (d), and test case 3 with a majority of 31.14% for option (b)).

Table 9 and Table 10 show the most selected option for the same subjects, but grouped by art-related background and the stated gender identity of the participants respectively. Grouping by participant information shows more detail about each group’s responses and eliminates the effect of the majority group’s dominance on the overall result.

In Table 9, the majority group is interested in art (56.82%), and the dominance of this group clearly affects the final results in the cases:

- For G-Dragon, two other groups chose option (a), and the majority chose option (b);
- For Barack Obama, all other groups chose option (c), and the majority chose option (d);
- For test case 1, two other groups chose option (b), and the majority chose option (a).

On the other hand, the majority group could not dominate in test case 3, although their choice was option (a).

In Table 10, the majority of the group is male (62.22%). We can see the dominance of this group in all cases, as the female group chose option (a) for all subjects. The reason why the female group chose more styles for all subjects is not clear. However, we can assume that females prefer artistic output or that the participants are mainly concerned with art.

## 5. Discussion

A crowd-sourcing survey was advertised for one month, conducted online, and sent to all users of the application. Of the total of 525 total views, 370 users completed the survey. We were able to analyze the performance of DaVinciFace using a scale of attitudes toward identity and style compromise that included subjects of different genders, races, and ages. The results of the survey are not to be expected. It turned out that the use of DaVinciFace with celebrities or even with non-celebrities where the user is not personally involved in the portraits allows more tolerance for the loss of important identity features while retaining more style. In addition, the audience of DaVinciFace is mainly interested in art, artists, or art students, which might justify the skew of results toward more style in general.

*Identity preservation*: When users were asked to recognize celebrities portrayed with DaVinciFace, Figure 8 shows that the most frequently recognized celebrity is Lucy Liu, followed by Freddie Mercury and then Roberto Benigni, although we can attribute these celebrities to the age majority of participants. The low percentage of recognition for young celebrities such as Billie Eilish is as expected, but we can say that DaVinciFace might have more recognition if it is a celebrity in general. The low recognition of Maria Sharapova as a celebrity in sports can also be explained by the fact that the audience is more interested in art. However, the case of Morgan Freeman is the most interesting, as he is internationally known and has a longer career. The reason for the low percentage of recognition could point to the bias of the DaVinciFace toward subjects of African descent.

*Identity/style trade-off*: The results of the other eight questions (Table 8, Table 9 and Table 10) show that participants generally chose to maintain the Da Vinci style even if it meant losing identity features. This is particularly evident when the subject is a Caucasian Female (Monica Bellucci, test cases 2 and 4) (see Table 8 and Table 9). However, further analysis based on the participant’s stated gender identity (Table 10) shows that the first option for Females retains most of the style, regardless of the subject’s characteristics. The reason for this bias toward option (a) (more style) cannot be determined, whether due to a bias of the model toward female subjects, due to the fact that Da Vinci’s works contained mostly female subjects, or due to the fact that the identity is somehow preserved in this particular case.

*Social perspective*: For individuals with darker skin tones (e.g., Barack Obama and test case 3), option (a) is not consistently preferred across all groups, as mentioned above. It is still crucial to maintain distinctive identity characteristics, even if stylistic nuances are gradually softened. This also applies to personalities such as Morgan Freeman. The reason for this—whether it is the loss of identity features (such as the lower eyelid, nose, and lip shape) or the fact that participants are unfamiliar with Da Vinci’s style, which is synonymous with such distinct features—is not yet clear. Furthermore, since the changes in skin color affect all subjects influenced by the style’s color palette, the effect could be more pronounced in those with darker skin tones. Further analysis is required to determine whether this bias stems from the training data used in StyleGAN2 or from the DaVinciFace application. In contrast, for the bearded man (e.g., Luciano Pavarotti and test case 1), option (a) is typically—but not always—preferred. Although Da Vinci’s style did not traditionally include a beard, participants often favored this style over more masculine features, especially the beard.

However, we can conclude that the highest bias of the model toward the reference photo is observed in people of African race according to our participants. A lower bias is observed for bearded males, while Caucasians and Asians, especially females, were also accepted by the participants with a less identity-preserving but more style-preserving option.

## 6. Conclusions

In this paper, we presented the exploration of the latent space of StyleGAN2 by analyzing it from the perspective of social features. We concluded that sparse vectors have a greater effect on these features. To evaluate our results, we conducted a survey that we sent to the users of DaVinciFace to collect their feedback, and we collected 360 responses. We demonstrate the analysis of these responses and find that the crowd-sourcing application maintains style even when identity or gender-specific features are lost, with the exception of African individuals.

The main limitation in generalizing these results is the subjective opinion of participants, especially if they are not related to or interested in art. Another known limitation is that surveys are best suited to show trends. In addition, the survey was only sent to users who have already tried DaVinciFace before, which can lead to a personal bias based on previous experiences. These results will be taken into account when designing the next version of the survey with new industry and research questions to appeal to a wider audience. Another approach to exploring the latent space is to use a reinforcement agent that aligns with the survey results to create the “perfect” portrait.

As a next step, we plan to develop strategies to improve the DaVinciFace features and mitigate biases in both the dataset and the corresponding AI application, especially biases affecting individuals with darker skin tones. We also want to compare different artistic styles to evaluate their impact on the accuracy of face recognition.

## Figures and Tables

**Table 1:** Examples of the original images of the subjects in the first row with the DaVinciFace portraits, using the default settings, for each in the second row.

Original Portraits	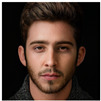	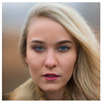	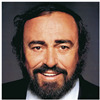	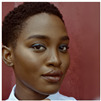	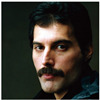	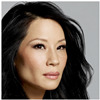
Default Settings of DaVinciFace	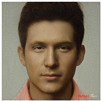	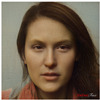	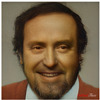	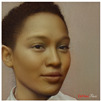	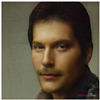	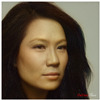

**Table 2:** The subjects of the survey were four different celebrities (Monica Bellucci, Luciano Pavarotti, G-Dragon, and Barack Obama) and four other non-celebrities test subjects. The subjects were selected to include different social categories, such as female and male, bearded and non-bearded men, light and dark hair and skin, as well as different races and age groups.

Monica Bellucci	Luciano Pavarotti	G-Dragon	Barack Obama
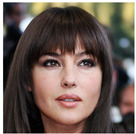	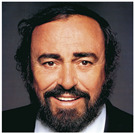	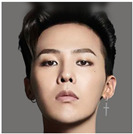	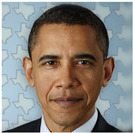
Test Case 1	Test Case 2	Test Case 3	Test Case 4
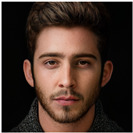	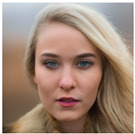	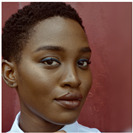	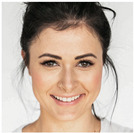

**Table 3:** ISOMAP reduction of the 18 vectors in two dimensions visualized by the scatter plot.

Vector 0	Vector 1	Vector 2
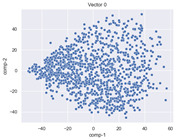	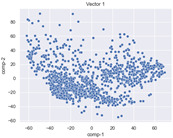	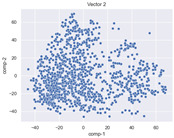
**Vector 3**	**Vector 4**	**Vector 5**
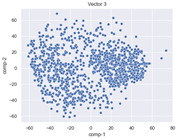	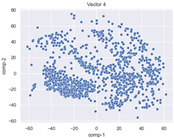	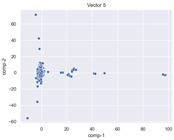
**Vector 6**	**Vector 7**	**Vector 8**
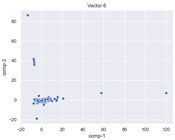	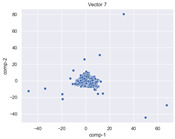	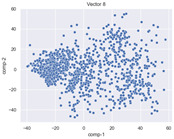
**Vector 9**	**Vector 10**	**Vector 11**
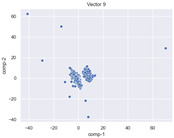	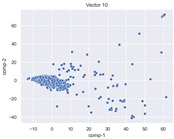	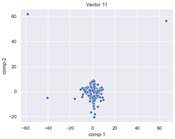
**Vector 12**	**Vector 13**	**Vector 14**
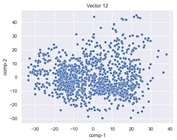	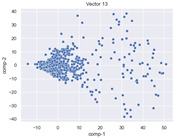	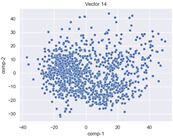
**Vector 15**	**Vector 16**	**Vector 17**
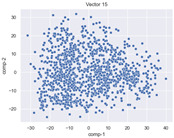	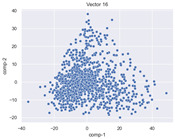	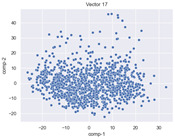

**Table 4:** ISOMAP reduction of the 18 vectors in two dimensions visualized by the kernel density estimate plot.

Vector 0	Vector 1	Vector 2
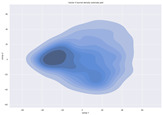	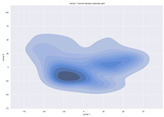	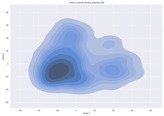
**Vector 3**	**Vector 4**	**Vector 5**
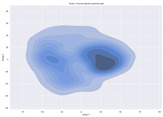	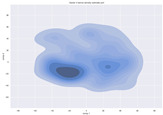	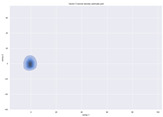
**Vector 6**	**Vector 7**	**Vector 8**
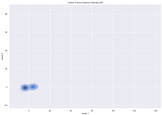	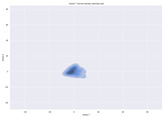	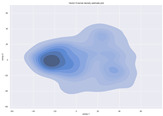
**Vector 9**	**Vector 10**	**Vector 11**
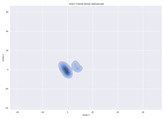	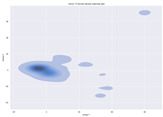	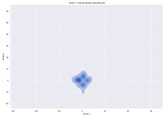
**Vector 12**	**Vector 13**	**Vector 14**
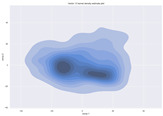	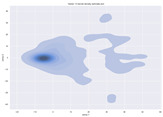	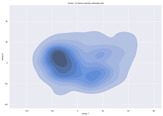
**Vector 15**	**Vector 16**	**Vector 17**
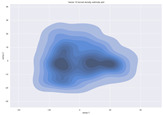	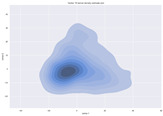	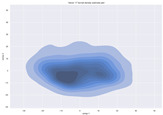

**Table 5:** ISOMAP visualization of the density of the 18 vectors, calculated by the mean Euclidean distance. The underlined numbers stand for a higher mean distance, which indicates a sparser distribution.

Vector 0	Vector 1	Vector 2	Vector 3	Vector 4	Vector 5
39.43	49.18	45.37	49.73	48.80	5.89
**Vector 6**	**Vector 7**	**Vector 8**	**Vector 9**	**Vector 10**	**Vector 11**
5.42	7.02	35.96	6.36	10.95	6.17
**Vector 12**	**Vector 13**	**Vector 14**	**Vector 15**	**Vector 16**	**Vector 17**
23.92	11.70	26.86	23.45	19.30	18.95

**Table 6:** Compare the results of the style transfer. From left to right: the subject image (original), the default settings, add vector 8, add vector 9, add vector 10, add vector 11.

Subject	Default settings	+Vector 8	+Vector 9	+Vector 10	+Vector 11
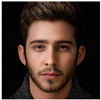	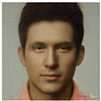	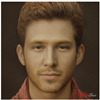	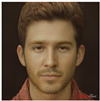	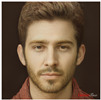	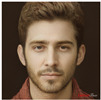
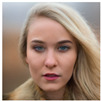	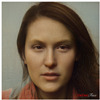	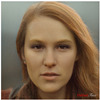	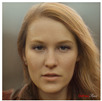	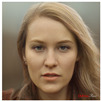	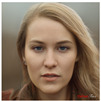
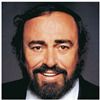	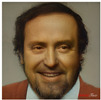	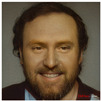	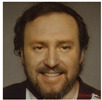	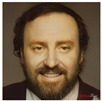	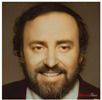
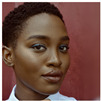	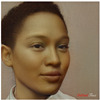	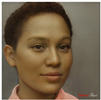	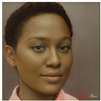	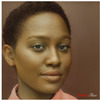	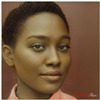
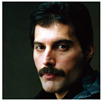	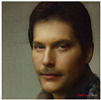	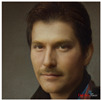	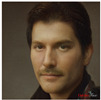	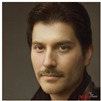	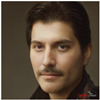
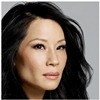	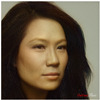	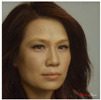	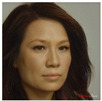	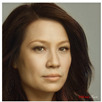	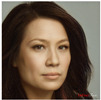

**Table 7:** The effect of vector 8 on identity and gender features on test cases 1, 2, and 5 further examples of bearded and blond-haired individuals to generalize our results. *First row:* the first 12 vectors are from the subject including vector 8; *second row:* the first 12 vectors are from the subjects, excluding vector 8.

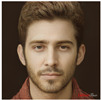	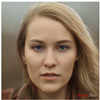	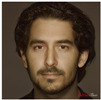	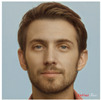	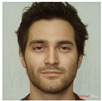	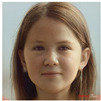	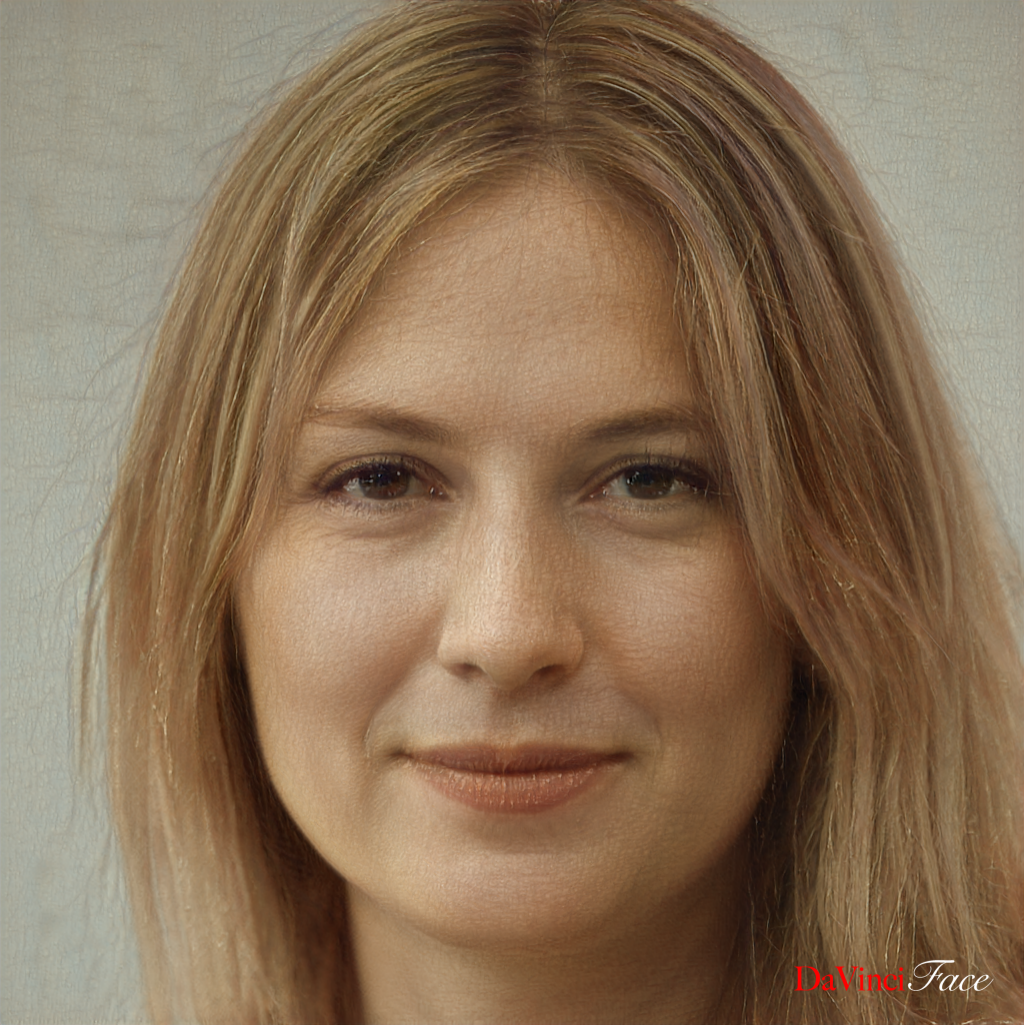
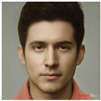	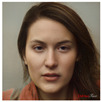	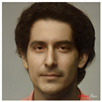	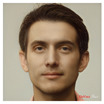	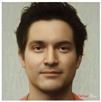	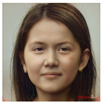	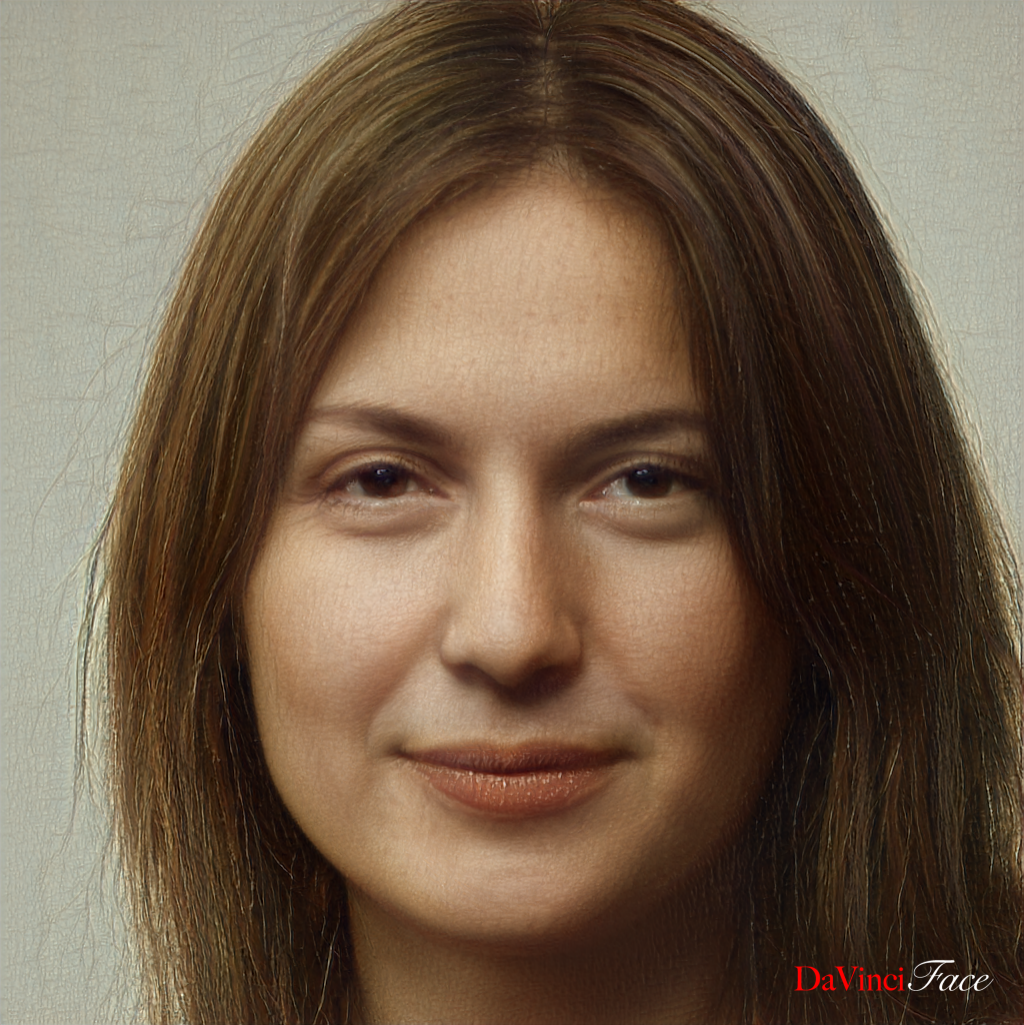

**Table 8:** Detailed statistics on the participants’ responses to the 8 survey questions, in bold is the highest percentage for each subject. The options are (a) default settings, (b) add to vector 8 from the original image, (c) add to vector 10 from the original image, (d) add to vector 11 from the original image.

Subject	Description ^1^	Total Responses	Option a (Default)	Option b (+Vector 8)	Option c (+Vector 10)	Option d (+Vector 11)
Monica Bellucci	Woman, Brunette	343	**67.93%**	10.79%	9.62%	11.66%
Luciano Pavarotti	Bearded man, Caucasian	349	**43.84%**	23.78%	15.19%	17.19%
G-Dragon	Man, Asian	346	35.84%	**38.15%**	13.01%	13.01%
Barack Obama	Man, African	350	26%	16.57%	28.57%	**28.86%**
Test case 1	Bearded man, Caucasian	346	**31.21%**	25.43%	22.83%	20.52%
Test case 2	Woman, blonde	352	**45.74%**	11.65%	23.30%	19.32%
Test case 3	Woman, African	167	30.54%	**31.14%**	18.56%	19.76%
Test case 4	Woman, Brunette	167	**57.49%**	20.96%	10.78%	10.78%

^1^ The descriptions of the subject’s images are our subjective opinions to categorize the subjects under diversity aspects.

**Table 9:** Detailed statistics of the answers to the 8 questions of the survey, grouped by art reference. The options are (a) default settings, (b) add vector 8 from the original image, (c) add up to vector 10 from the original image, (d) add up to vector 11 from the original image.

Art-Related	Monica	Luciano	G-Dragon	Barack	Test Case 1	Test Case 2	Test Case 3	Test Case 4
Professional artist	a (65%)	a (43%)	a (43%)	c (37%)	a (32%)	a (49%)	c (31%)	a (45%)
Art student	a (61%)	a (58%)	b (44%)	c (32%)	b (56%)	a (37%)	b (50%)	a (70%)
Interested in art	a (72%)	a (44%)	b (41%)	d (32%)	a (32%)	a (47%)	a (39%)	a (59%)
Not related to art	a ( 64%)	a (37%)	a (38%)	c (34%)	b (32%)	a (41%)	b (33%)	a (60%)

**Table 10:** Detailed statistics of the participants’ answers to the 8 questions of the survey, grouped by gender. The options are (a) default settings, (b) add vector 8 from the original image, (c) add up to vector 10 from the original image, and (d) add up to vector 11 from the original image.

Gender	Monica	Luciano	G-Dragon	Barack	Test Case 1	Test Case 2	Test Case 3	Test Case 4
Female	a (72%)	a (52%)	a (39%)	a (28%)	a (29%)	a (53%)	a (40%)	a (64%)
Male	a (66%)	a (39%)	b (37%)	d (30%)	a (32%)	a (42%)	b (31%)	a (53%)

## Data Availability

The data used in this work are unavailable due to privacy and ethical restrictions.

## Abbreviations

The following abbreviations are used in this manuscript:

GANs	generative adversarial networks
VAEs	variational autoencoder
ISOMAP	isometric mapping
t-SNE	t-distributed stochastic neighbor embedding
UMAP	uniform manifold approximation and projection
AI	artificial intelligence
